# Evaluating Web-Based Care for Mental Health and Substance Use Issues for Lesbian, Gay, Bisexual, Transgender, Queer, Questioning, and 2-Spirit Youths in the Context of the COVID-19 Pandemic: Community-Based Participatory Research Study

**DOI:** 10.2196/44292

**Published:** 2023-11-17

**Authors:** Michael Chaiton, Rachel Thorburn, Emily Chan, Ilana Copeland, Chieng Luphuyong, Patrick Feng

**Affiliations:** 1 Ontario Tobacco Research Unit Dalla Lana School of Public Health University of Toronto Toronto, ON Canada; 2 Institute of Medical Science Temerty Faculty of Medicine University of Toronto Toronto, ON Canada; 3 Institute for Mental Health Policy Research Centre for Addiction and Mental Health Toronto, ON Canada; 4 Applied Psychology and Human Development Ontario Institute for Studies in Education University of Toronto Toronto, ON Canada; 5 Factor Inwentash School of Social Work University of Toronto Toronto, ON Canada; 6 Ontario College of Art & Design Toronto, ON Canada; 7 Institute of Health Policy, Management, and Evaluation University of Toronto Toronto, ON Canada

**Keywords:** web-based care, COVID-19, LGBTQ2S+, youth, mental health, substance use, web-based, care, quality of care, design, communication, policy, model, cost, service

## Abstract

**Background:**

Mental health (MH) and substance use (SU) care supports are often difficult to access for the lesbian, gay, bisexual, transgender, queer, questioning, and 2-spirit (LGBTQ2S+) population. There is little known on how the shift to web-based care has affected and changed the experiences of LGBTQ2S+ youths within the MH care system.

**Objective:**

This study sought to examine how web-based care modalities have affected access to care and quality of care for LGBTQ2S+ youths seeking MH and SU services.

**Methods:**

Researchers used a web-based co-design method to explore this population’s relationship with MH and SU care supports, focusing on the experiences of 33 LGBTQ2S+ youths and their relationship with MH and SU supports during the COVID-19 pandemic. A participatory design research method was used to gain experiential knowledge of LGBTQ2S+ youths’ lived experience with accessing MH and SU care. Thematic analysis was used to examine the resulting audio-recorded data transcripts and create themes.

**Results:**

Themes related to web-based care included accessibility, web-based communication, provision of choice, and provider relationship and interactions. Barriers to care were identified in particular for disabled youths, rural youths, and other participants with marginalized intersecting identities. Unexpected benefits of web-based care were also found and emphasize the idea that this modality is beneficial for some LGBTQ2S+ youths.

**Conclusions:**

During the COVID-19 pandemic, a time where MH- and SU-related problems have increased, programs need to reevaluate current measures so that the negative effects of web-based care modalities can be reduced for this population. Implications for practice encourage service providers to be more empathetic and transparent when providing services for LGBTQ2S+ youths. It is suggested that LGBTQ2S+ care should be provided by LGBTQ2S+ folks or organizations or service providers who are trained by LGBTQ2S+ community members. Additionally, hybrid models of care should be established in the future so that LGBTQ2S+ youths have the option to access in-person services, web-based ones, or both as there can be benefits to web-based care once it has been properly developed. Implications for policy also include moving away from a traditional health care team model and developing free and lower-cost services in remote areas.

## Introduction

Lesbian, gay, bisexual, transgender, queer, questioning, and 2-spirit (LGBTQ2S+) youths and young adults are at higher risk of developing several mental health (MH) conditions, including suicidality and substance use (SU)–related risks and harms [1,2]. Prepandemic, sexual and gender minority youths have been shown to experience higher rates of emotional distress and discrimination due to the heteronormativity of medical and social service systems along with stigma and perceived homophobia from their familial and social support networks [1]. The effects of this discrimination have been exacerbated by the recent COVID-19 pandemic. Craig et al [3] found that “pandemics create a perfect storm for vulnerable populations by exacerbating existing stressors and eliminating access to services that are urgently needed.” A Canadian study conducted by Egale Canada and INNOVATIVE research group reported that a higher proportion (42%) of LGBTQ2S+ participants was experiencing significant negative impacts on their physical health, MH, and quality of life during the pandemic, as compared to the general public (30%) [4].

During this time, LGBTQ2S+ youths and young adults are especially vulnerable because they are having to quarantine in potentially hostile family environments, which can increase rates of domestic abuse and decrease access to web-based services [5-8]. Physical distancing measures or “stay-at-home” orders to reduce community transmission of COVID-19 have worsened *distal minority stress* for LGBTQ2+ youths, meaning external stressors created through discriminatory events such as family rejection due to one’s gender or sexual identity [7]. The Trevor Project’s third annual survey taken during the course of the pandemic found that “42% of LGBTQ2S+ respondents seriously considered attempting suicide in the past year, including more than half of transgender and non-binary youth” [9].

During the COVID-19 pandemic, in-person MH and SU services for LGBTQ2S+ youths shifted to offer web-based care [3]. Anonymous text-based platforms such as TrevorSpace and Discord Servers have also been providing web-based support to LGBTQ2S+ youths, both before and during the pandemic [6]. Some interventions such as cognitive behavioral therapy and supportive peer groups have shown to be effective when delivered on the web [1,10]. However, other treatment modalities have proven unsuccessful when delivered in a web-based format [5]. More research is needed to understand the impact of web-based care in this population.

There exists a large body of research that calls on policy makers and service providers to improve LGBTQ2S+ youths’ and young adults’ access to web-based care because of the unique gaps that have arisen from the COVID-19 pandemic [11]. Our research seeks to improve access to MH and SU care services for LGBTQ2S+ youths and young adults in the context of the pandemic. This paper examines how web-based care modalities introduce or exacerbate challenges for LGBTQ2S+ youths to access appropriate MH and SU care supports.

## Methods

### Study Design

This study is embedded in a larger research program entitled “Sexual & Gender Minority Youth Access to Services in Mental Health During the COVID-19 Pandemic” (S.M.A.S.H COVID), a project based out of the Centre for Addiction and Mental Health, a psychiatric teaching hospital located in Ontario, Canada. A previous analysis by the research team using survey data from 1404 Canadian LGBTQ2S+ youths indicated that 51.8% of respondents identified a need for MH or SU support during the COVID-19 pandemic but experienced barriers to accessing care [12]. S.M.A.S.H COVID was a community-based participatory research project that sought to better understand the experiences of LGBTQ2S+ youths who are facing barriers to accessing MH and SU care services in the context of the COVID-19 pandemic. The project was guided by the conceptual framework of grounded theory. Grounded theory involves the construction of hypotheses or theories through the collection or analysis of data [13]. It is typically used in qualitative social sciences research to understand social processes from the perspective of individuals involved in those processes [13]. Grounded theory was chosen for S.M.A.S.H COVID because the research team wanted to understand the barriers LGBTQ2S+ youths were experiencing from the perspectives of the youths themselves and to listen and learn from their experiences and ideas rather than come in with preconceived ideas about the issues participants faced. Every step of the research process, from conception to analysis, included LGBTQ2S+ youths on the research and facilitation team.

S.M.A.S.H COVID used a design charrette model or a series of intensely focused, multiday sessions with activities designed to collaboratively elucidate barriers to care and create realistic and achievable solutions [14]. The design charrette model was chosen for this project because it is a collaborative approach to generating sustainable solutions to complex problems [14]. The specific research activities within the design charrette were designed by graduate students from OCAD University who identified as LGBTQ2S+ youths or allies. Eight facilitators led the sessions, including 5 LGBTQ2S+ youths and 3 allies. Facilitators were members of the research team, OCAD graduate students, or youth engagement facilitators from Centre for Addiction and Mental Health.

In total, 33 LGBTQ2S+ youths aged 16-29 years and located in Ontario or Quebec participated in the program, held digitally on Webex, over 3 sessions in June 2021. In total, 33 was chosen as our sample size based on recommendations for design charrettes and thematic analysis that suggest picking a sample size that is small enough to manage the data and large enough to demonstrate patterns, with some sources indicating the ideal size should be between approximately 30 and 40 [14-16]. See Table 1 for participant demographics collected through a short survey completed by participants prior to beginning the sessions. Note that this sample was previously discussed and described in [17]. All participants had accessed or tried to access MH or SU care during the COVID-19 pandemic. Participants were not asked to disclose information about the specific types of support they had sought. Participants were recruited through email invitation to the Public Health Agency of Canada 2SLGBTQI+ Campaign on Commercial Tobacco Use and Its Culture cohort network and by referent recruitment through research team members.

This analysis focuses on the first session, where participants engaged in an activity called “journey mapping.” *Journey mapping* is a method for visualizing the process that a user goes through in the course of accomplishing a goal [18]. Originally developed for user-experience and customer-based research, it has been adopted in other sectors, including health care [18]. Journey mapping was used as a safe and low-threshold engagement activity for participants, where conversation was a mechanism for group knowledge sharing.

**Figure 1:**
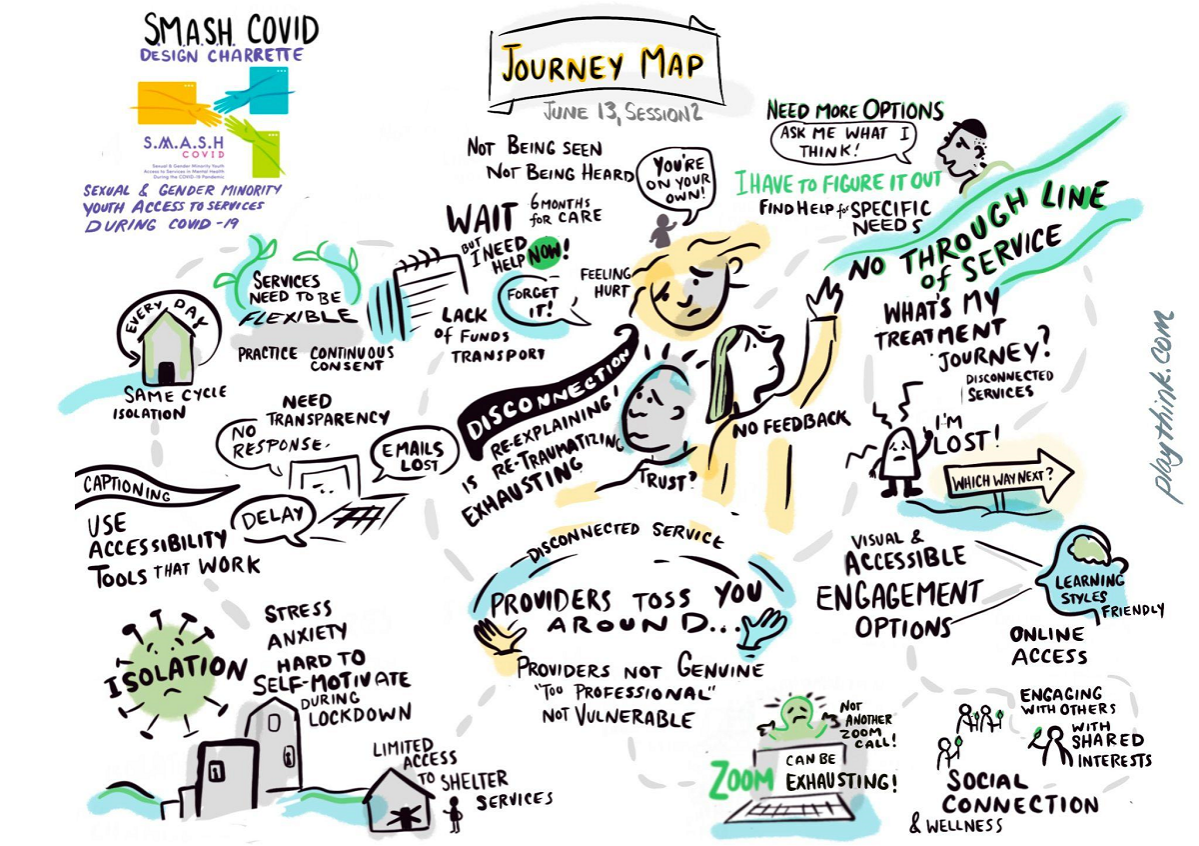
An example of one of the journey maps created. S.M.A.S.H. COVID: Sexual & Gender Minority Youth Access to Services in Mental Health During the COVID-19 Pandemic.

Participants were split into 4 groups for a guided hour of discussion on the research question: what barriers exist for LGBTQ2S+ youths and young adults to access identity-affirming mental health care during the COVID-19 pandemic? As participants spoke, a graphic illustrator visually mapped discussed journeys, creating a tangible representation of participants’ shared experiences for each of the 4 groups (for an example of one of the journey maps created, see Figure 1).

All journey mapping sessions were audio-recorded. Braun and Clarke’s [19] thematic analysis was used to analyze the recorded data. Together, 5 members of the research team listened to the audio recordings, including 2 team members who identify as LGBTQ2S+ youths. The researchers then used thematic analysis to identify prominent themes in the data. The audio recordings were then transcribed, and the data were reviewed again by 3 research team members (including 1 LGBTQ2S+ youth) to reassess the appropriateness of each theme and identify relevant quotes. The final draft of this paper was edited and approved by an LGBTQ2S+ youth member of the research team.

Based on this analysis, research team members also created a vignette to illustrate key themes from the journey mapping exercise. The vignette is presented below, as an introduction to this study’s results.

**Table 1:** Participant demographics.

Demographics		Participants, n (%)
**Gender identity**		
	Agender	2 (6)
	Genderfluid	3 (9)
	Genderqueer	4 (12)
	Man	2 (6)
	Nonbinary	8 (24)
	Trans man	3 (9)
	Trans woman	1 (3)
	Two-spirit	1 (3)
	Woman	9 (27)
**Sexual identity**		
	Asexual	1 (3)
	Bisexual	6 (18)
	Gay	3 (9)
	Lesbian	4 (12)
	Pansexual	4 (12)
	Queer	10 (30)
	Questioning	1 (3)
	Straight or heterosexual	2 (6)
	Two-spirit	2 (6)
**Lived in Canada**		
	From birth	27 (82)
	More than 10 years	2 (6)
	1-10 years	4 (12)
**Highest level of education**		
	Some high school	8 (24)
	High school diploma	13 (39)
	More than high school	11 (33)
**Employment status**		
	Employee	7 (21)
	Self-employed	2 (6)
	Working unpaid	1 (3)
	Student	12 (36)
	Long-term sick or disabled	3 (9)
	Unemployed	4 (12)
	Unemployed owing to the COVID-19 pandemic	4 (12)
**Person living with a disability**		
	Yes	18 (55)
	No	15 (45)
**Race**		
	African Canadian or American	1 (3)
	Black	4 (12)
	Caribbean	4 (12)
	East Asian	1 (3)
	First Nations	3 (9)
	Latin American	1 (3)
	Métis	2 (6)
	Middle Eastern	2 (6)
	Multiracial or mixed	3 (9)
	South Asian	4 (12)
	White	8 (24)

### Ethics Approval

This study was approved by The Centre for Addiction and Mental Health Research Ethics Board (approval 039/2021).

## Results

### Overview

Participants spoke about their experiences in accessing MH and SU care during the pandemic. While the research team initially expected participants to describe their journeys accessing care, participants described their experiences more as a series of disconnected events. While each participant’s experience was unique, common themes from the discussion were identified by the research team, which are synthesized in Textbox 1.

Overall, 4 themes specific to LGBTQ2S+ youths seeking web-based care were identified. In addition to the 4 web-based themes, we identified other general themes that were not specifically related by participants to web-based care but were prominent points of discussion that participants identified as important barriers to accessing MH and SU support. We elaborate on these themes below.

**Vignette of participant experiences accessing care.**

John is an 18-year-old university freshman who started school in 2019. Due to the COVID-19 pandemic in March 2020, all of John’s classes became web-based. John had always struggled with feelings of anxiety and depression in the past; however, these feelings intensified during the pandemic. John decided to speak with a counselor about his experience, though he was nervous due to being misgendered by counselors he had seen in the past. When he called the counseling office, John was not able to speak with anyone directly and was told to leave his contact information and that someone would get back to him (theme: web-based communication). After waiting for 2 weeks with no callback, John emailed the office to follow-up. A week later, the receptionist from the counseling office finally returned John’s email. At this point, John had waited 3 weeks for an initial response from the clinic (themes: web-based communication and accessibility). They told that there would be a 3-month wait for services, that they would not be able to choose their counselor, and that the appointments would be held digitally (theme: provision of choice). John was nervous because his good counseling experiences in the past were due to the relationship he was able to build with his counselor in person. However, he was relieved that it would be easy for him to attend web-based sessions since the counseling office would be difficult for him to access without a car (theme: accessibility).

After waiting for 3 months, John attended their appointment and was repeatedly misgendered by his counselor. The internet also cut out multiple times during the appointment, and John lost 10 minutes of his 50-minute session due to technical issues (themes: web-based communication and accessibility). John felt that he was not able to connect with his counselor over the screen in the same way he had previously with his in-person counselor (theme: provider relationship and interactions). John felt defeated and did not end up making another appointment.

### Themes Specific to Web-Based Care

#### Theme 1: Accessibility

Overall, participants reported that web-based care led to better accessibility to services than that before the COVID-19 pandemic, both in the sense of being physically easier to access and also because many services reduced or waived their fees for web-based care options:

There's these kinds of things that are online now, although they're more accessible, which is wonderful. And some, some of the fees are even being waived, which is so wonderful, too.

LGBTQ2S+ youths from rural communities shared that they traditionally experience reduced access to in-person care and often have to travel far due to a lack of services in their area. Rural youths reported that the move to web-based care reduced barriers associated with traveling into the city for in-person services, allowing them more choices in providers, more options for specialized services, and greater access to service providers who were adequately trained to work with gender and sexually diverse youths.

Disabled youths also reported that web-based care resulted in better access to services than they had experienced before the pandemic. A facilitator noted the following comments made during the conversation by a participant through text chat:

They've been doing online stuff for all their life…so saying something that's online they are accustomed to. And…that worked for them… they were saying it's also been way more exhausting to not have online options, and COVID is honestly the most accessible than it’s ever been in (their) life.

Despite the overall benefit of web-based care for accessibility, disabled youths also noted that web-based accessibility tools need to work for those relying on them. For example, web-based modalities often offer closed captions; however, this service is only helpful when the captions are clear and coherent. For some neurodivergent youths, web-based care also posed unique barriers. Particularly, phone and email services made communication challenging for participants who rely heavily on visual social cues.

Finally, participants noted that web-based services are not accessible to all youths. Specifically, they mentioned that some youths do not have access to the internet or technology devices to access web-based services and that many LGBTQ2S+ youths and young adults do not have a safe or supportive space to access web-based care:

Due to COVID they have just begun doing counseling virtual…but like some folks have moved back home with their parents or are living in rooming houses. For me, I live with my parents, and I'd say that like privacy is a big concern for me. So in some ways, yes, things are more accessible, but there's also a lot of limitations and like concerns regarding how you access things.

Overall, the move to web-based care increased the accessibility of services compared to in-person care, especially for specific subpopulations that face barriers to in-person care, such as rural youths and disabled youths. However, barriers to web-based care were also identified, including challenges navigating less social cues, lack of internet access, and lack of safe or supportive spaces to access web-based care.

#### Theme 2: Web-Based Communication

Participants reported difficulty communicating and building rapport on the web or over the phone. During email communication, many participants did not feel heard over email and expressed issues with timely responses from providers. When email communication did occur, it felt more formal and impersonal and not as organic or effective as a “natural conversation.” Participants noted that the nature of email as opposed to an in-person interaction meant that they had to self-motivate more to continue to reach out and did not receive feedback right away, which some found challenging.

There's no feedback immediately about what you're talking about. You have to like, think about everything while you're writing that email or while you're getting ready for something. Because when you're with somebody and talking to someone, like naturally, you think of things as it happens. Whereas if you're writing an email, you have to kind of get everything out, and then like, go over it, make sure you've gotten all your points down.

However, some participants benefited from email communication. For example, when having gender-affirming surgery, a participant reported that they preferred being able to email their provider about the surgery and “just being able to disconnect, write it down, and send it off” without worrying about what the provider thought about them.

Phone anxiety was identified as an obstacle to effective care for services that required phone conversations for registration or service provision. Regarding both email and phone conversation, some participants noted that they rely on body language as a communication aid, and the ability to read facial expressions and establish eye contact is not present in the forms of web-based modalities such as emails and phone calls. This was especially challenging for participants who identified as neurodivergent.

With video service modalities, participants felt it was harder to be vulnerable on Zoom. Screen fatigue and exhaustion were common issues that arose, especially for participants who also had work or school on the web. The exhaustion and stress of web-based communication resulted in low motivation to access web-based services for some participants, who reported that the web-based format did not feel as effective as in-person services. However, other participants noted that in-person services could be much more exhausting in different ways and that web-based services were much more accessible to them. In general, participants felt that technology-mediated communication was less personal and identified a lack of human connection with provider interactions during the pandemic as compared to in-person interactions.

#### Theme 3: Provision of Choice

Another recurring theme noted by participants was the lack of care options presented to them. Participants described the lack of options, especially during the COVID-19 pandemic, as inflexible and unaccommodating and stressed that this one-size-fits-all approach did not work in the context of many of their unique circumstances:

There's like this whole one size fit all kind of thing or, you know, we think this will work for all these people. And then within gender and sexual diversity, there's a whole other, you know, what is affirming care? To who? why?, like, for which identities does that make sense?

Youths described that it was important for the success of their care to be given options such as being able to choose their therapy format (eg, web-based or in-person), to choose their own care provider who could best meet their individual needs, and to be given options about what treatment modalities they felt fit their situation best. Overall, they felt care providers should be taking greater care to consult them in their own treatment process, in order to meet their individual needs.

I wish there would have been, they would have asked me what I think would have helped me or based on like, my culture or my personality, they would have asked me what I think would have been best, instead of just telling you this is what I'm going to do and it will make them better when it did not help.

#### Theme 4: Provider Relationship and Interactions

Participants experienced several issues while accessing care related to provider relationships and interactions including a perceived lack of provider warmth or compassion from web-based care. Participants described a lack of basic empathy or compassion from providers, which made youths feel like just a number or “someone’s problem” when trying to access care. They felt that providers did not care about them and “like they don't want to know your problems,” and their concerns went unheard as a result. This was reflected in situations such as providers being slow to respond to urgent situations and showing up late or not adequately preparing for client sessions.

I've had contacted with therapists and you know, other support people, via emails via texting, phone calls, and it just feels impersonal. And it's almost like they don't really hear you will have time for you. Because you're just another person on a screen, you're just another email.

Another issue identified was a need for a consistent provider relationship, a challenge made even more difficult by the shift to web-based care. Multiple participants said building relationships with providers was important because it was harder to share sensitive information with service providers without mutual trust and established rapport—a process that was more difficult digitally. Participants said that being “tossed around” from one provider to another made it hard to build relationships and trust and that this made the care-seeking process inefficient. Participants felt that their interactions should be a bond-building process, but instead often felt like web-based interactions were transactional, and that providers were trying to quickly end sessions. They wanted service providers to take the time to build a genuine connection despite the challenges of web-based care. This was further complicated by issues such as a limit to the number of interactions they could have with a provider.

Um, one thing that I know is that when you do connect with people during the pandemic, it's very professional, and it's not as it's not as like, vulnerable as it would be in person because you don't have that bond and connection with people on like, a base level. It's, it feels less because you know, that both people are just sitting, looking at the screen and you know, you, you lose something in that.

### Systemic Issues not Specifically Related to Web-Based Care

Participants also spoke to longstanding systemic problems in the provision of MH and SU care for LGBTQ2S+ youths and young adults that affected their journey through the MH system that was independent of web-based care. Although these issues are not pandemic-specific, they are worth noting as ongoing barriers being experienced by gender and sexually diverse youths and young adults who seek affirming care. Furthermore, responses from participants suggest the pandemic may have worsened many of these access-to-care issues.

One issue was provider competency. This included the need for informed, affirming, and educated providers from diverse backgrounds who were understanding of the needs of the LGBTQ2S+ community and intersecting identities. Training should be required for providers to LGBTQ2S+ youths to have a level of care that is consistent with the needs of service users, particularly LGBTQ2S+ and Black, Indigenous, and people of color youths. Service providers in rural areas were identified as being in particular need of this kind of competency, although it was identified as an issue across geographic locations. Participants identified that a lack of provider competency resulted in experiences of discrimination when trying to access care. This was particularly salient in the discussion for trans participants, who shared painful experiences of being misgendered or deadnamed by providers. Participants reported that these experiences of discrimination were detrimental to their MH. Relatedly, participants also identified the need for safer opportunities to provide feedback to providers and for training programs to be followed up with systems of accountability to ensure providers were incorporating their learnings into their practice. Youths wanted to be at the center of their own care and for the therapy process to be one of continuous feedback and consent.

Wait times were reported as a significant access barrier for many participants, especially within rural communities. Participants mentioned the lack of support and transparency during wait times as causing major delays that create disconnects in care and hinder progress along their care journeys. It was noted that the pandemic created further delays to existing waitlists.

Navigating services that were web-based or not can be overwhelming for LGBTQ2S+ youths. MH and SU care often involve multiple providers, and this can be challenging to manage. With limited options, it is hard for youths to know where to start and what is available. For example, one participant mentioned ambiguous labeling like “youth wellness” as not indicative of what kind of services are offered and another said that they were looking for recovery support but were not sure what that should look like (eg, abstinence vs harm reduction). Other difficulties brought up by participants included the need to “self-motivate” to access care, the inability to be present and show up for oneself during a therapy session, trying to meet various learning needs within group settings, and the anxiety of making phone calls after discharge to get follow-up support.

Lack of continuity of care was a significant theme. Participants do not experience a journey—instead, they experience isolated moments of care that are not connected. Despite Ontario’s transition into a “health care team” model, participants viewed the care for LGBTQ2S+ youths as “siloed.” The lack of communication among providers (even in the same care team) burdened participants who had to build new relationships and re-explain their personal situation with strangers.

One participant noted on systems navigation:

I'd also say like, like referrals, or just like setup in general…that whole process can be very discouraging, debilitating, exhausting, and lengthy. Right, and you have to get a GP before you're able to get a psychiatrist, you have to go through all these steps…., and sometimes these things take months and months at a time. And so by the point that you have one step completed, so much has happened to you, and that time, and it's like yeah, I needed your help then and it's almost like I don't even I don't even want anymore.

## Discussion

### Principal Findings

The purpose of this study was to answer the question: how do web-based care modalities introduce or exacerbate challenges for LGBTQ2S+ youths to access appropriate MH and SU care supports? Using a web-based co-design method, researchers were able to engage with youths to better understand the barriers to web-based care. The primary set of themes was specific to web-based care and included accessibility, web-based communication, provision of choice, and finally provider relationship and interactions. More general barriers were also identified, including disparities in access, wait times, systems navigation, and continuity of care.

This study is one of the first to highlight the implications of COVID-19 on access to care for this population. Although all youths are affected by many of these barriers, previous research has established that existing social-structural inequalities are being exacerbated by the COVID-19 pandemic, and findings show that LGBTQ2S+ youths are disproportionately affected by this shift to the pandemic-related reliance on web-based care [5]. Supporting this, participants reported that the nature of informal and formal support has changed leading to consequences such as low motivation, increased screen fatigue, and phone anxiety. Furthermore, participants suggested that some LGBTQ2S+ youths might not have access to technological devices and that the ones who could access them may not have a safe space to do so, leading to further disparities when attempting to access MH and SU services.

Previous research indicates that using web-based care modalities and telehealth can be beneficial if implemented appropriately [20,21], and it was evident that participants did have more access to opportunities and resources through technology and digitally accessible services. The outcomes of this study add to a long-standing body of literature that advocates for the development of web-based care, enabling care seekers to choose which method (web-based, in-person, or hybrid format), treatment (affirmative CBT and peer-support groups), and provider best align with their personal values and perspectives [20,21]. Providers should invest in both web-based care and in-person services so both choices of care are available.

There are a few limitations of this study that are important to note. Because this study follows a unique qualitative design charrette research design, the findings were very experiential. Therefore, there should be discretion when generalizing this study’s findings to broader LGBTQ2S+ populations, as the sample size was limited. A related limitation is that although the sample used was appropriate for the research question, there could be more opportunities to increase the diversity of ethnocultural knowledge and experience. For instance, this study only engaged with participants who spoke English, causing a language barrier for potential participants. While the research team did maximize diversity among its English-speaking participants, more could have been done to engage with non-English speakers and LGBTQ2S+ youths who speak English as a second language.

Another complication is that this study was conducted on a web-based platform, and the sample excludes those who were not able to access technological devices and the internet. This normally includes LGBTQ2S+ youths who are homeless, in foster care, or live in poverty [7]. As this is an especially vulnerable subpopulation of LGBTQ2S+ youths (especially during the COVID-19 pandemic), including these perspectives would have made the sample size more inclusive. Furthermore, researchers and participants identified internet connection issues as another technological barrier. Some participants were not able to fully access the program due to tenuous internet connections, interfering with their ability to contribute. In the future, a hybrid model of research (web-based and in-person design) could be used to include more youths and increase the accessibility of the study.

Areas of future research could include more community stakeholders, such as service providers, so that more intersectional research on LGBTQ2S+ youths can be carried out. Involving stakeholders can lead to heightened knowledge mobilization into broader society so that mainstream MH and SU organizations can begin to work more with and for this population. Using community-based participatory action research for future studies could use community service providers and young people as key stakeholders, encouraging a greater understanding of the issues and more direct policy implementation.

Additionally, research should be conducted on barriers to access for LGBTQ2S+ adults during the COVID-19 pandemic. This would allow researchers to contrast the results between LGBTQ2S+ youths and adults and observe age, generational factors (eg, millennials vs baby boomers), and potentially technological literacy as factors that can influence these barriers. Research on LGBTQ2S+ adults can contribute to developing shared realities between the entire LGBTQ2S+ population during the pandemic and suggest cross-generational themes that arise when accessing web-based care.

As mentioned earlier, this study has an evident bias that neglected to recruit LGBTQ2S+ youths who did not have access to the internet, technological devices, a safe environment to go to, and those who did not speak English. Future designs need to incorporate the perspectives of youths outside these parameters as it is likely that these subpopulations are experiencing this shift to web-based care more so than the one that was just studied. Web-based interventions and services need to be able to accommodate all LGBTQ2S+ youths as we continue to shift into the era of web-based care.

### Conclusions

The purpose of this study was to examine the experiences of LGBTQ2S+ youths accessing web-based MH and SU care during the COVID-19 pandemic. Results suggest that although some youths benefited from the increased accessibility of web-based care, many found web-based care to introduce barriers of its own, such as issues communicating and connecting with service providers digitally. Additionally, challenges such as internet connection and needing a safe space at home to access care were identified. Existing issues such as gender and sexual identity discrimination, systems navigation, and waitlists for care were described as being exacerbated by the abrupt shift to web-based care. Participants stressed the need for more choices in their own care to individualize it to their needs, including choice of provider, modality (web-based or in person), and type of therapy.

The implications of this work are that service providers and the organizations they work for should strive to offer as many options as possible to clients to suit their needs, especially for groups such as LGBTQ2S+ youths who are at increased risk of experiencing systemic barriers and discrimination. Although web-based care has benefits and can increase accessibility for some youths, it presents barriers for others. Offering both in-person and web-based modalities can help clients choose the format that works best for them. Additionally, providers should strive to educate themselves on best practices when working with LGBTQ2S+ youths, in order to prevent issues such as discrimination that are exacerbated by the shift to web-based care.

## Abbreviations

LGBTQ2S+: lesbian, gay, bisexual, transgender, queer, questioning, and 2-spirit
MH: mental health
S.M.A.S.H. COVID: Sexual & Gender Minority Youth Access to Services in Mental Health During the COVID-19 Pandemic
SU: substance use
